# Nanostructured Surfaces and Detection Instrumentation for Photonic Crystal Enhanced Fluorescence

**DOI:** 10.3390/s130505561

**Published:** 2013-04-26

**Authors:** Vikram Chaudhery, Sherine George, Meng Lu, Anusha Pokhriyal, Brian T. Cunningham

**Affiliations:** 1 Micro and Nanotechnology Laboratory, Department of Electrical and Computer Engineering, University of Illinois, 208 North Wright Street, Urbana, IL 61801, USA; E-Mails: vikram.chaudhery@gmail.com (V.C.); menglu@iastate.edu (M.L.); 2 Micro and Nanotechnology Laboratory, Department of Bioengineering, University of Illinois, 208 North Wright Street, Urbana, IL 61801, USA; E-Mail: sgeorge4@illinois.edu; 3 Micro and Nanotechnology Laboratory, Department of Physics, University of Illinois, 208 North Wright Street, Urbana, IL 61801, USA; E-Mail: pokhriy1@illinois.edu

**Keywords:** Photonic crystal, fluorescence, nanostructured surface, nanolithography, fluorescence microscopy, DNA microarray, biomarker, cancer diagnostics

## Abstract

Photonic crystal (PC) surfaces have been demonstrated as a compelling platform for improving the sensitivity of surface-based fluorescent assays used in disease diagnostics and life science research. PCs can be engineered to support optical resonances at specific wavelengths at which strong electromagnetic fields are utilized to enhance the intensity of surface-bound fluorophore excitation. Meanwhile, the leaky resonant modes of PCs can be used to direct emitted photons within a narrow range of angles for more efficient collection by a fluorescence detection system. The multiplicative effects of enhanced excitation combined with enhanced photon extraction combine to provide improved signal-to-noise ratios for detection of fluorescent emitters, which in turn can be used to reduce the limits of detection of low concentration analytes, such as disease biomarker proteins. Fabrication of PCs using inexpensive manufacturing methods and materials that include replica molding on plastic, nano-imprint lithography on quartz substrates result in devices that are practical for single-use disposable applications. In this review, we will describe the motivation for implementing high-sensitivity fluorescence detection in the context of molecular diagnosis and gene expression analysis though the use of PC surfaces. Recent efforts to improve the design and fabrication of PCs and their associated detection instrumentation are summarized, including the use of PCs coupled with Fabry-Perot cavities and external cavity lasers.

## 1. Background and Motivation

Biological assays based upon fluorescence detection are the cornerstone of applications that include molecular diagnostics, gene expression microarrays, genome sequencing, protein-protein interaction screening, cell imaging, and many others [1]. The ability to couple a wide variety of fluorescent materials such as organic dyes, fluorescent proteins, and quantum dots with biological molecules and cells has revolutionized our ability to understand complicated biological phenomena, and to selectively detect biological targets within complex media. Typical fluorescence-based assays include determining how much of a specific biomolecule is present in a test sample and visualizing how molecules function within cells. A vast array of labeling materials, illumination sources, and detection instruments have been developed for labeling m-based assays that enable these methods to achieve detection of disease biomarker proteins in serum at concentrations below 1 pg/mL, to perform sub-diffraction limit imaging, and to perform highly multiplexed biomolecluar interaction analysis with throughput exceeding millions of individual locations on a single chip [2,3]. Fundamentally, fluorescence is an electromagnetic and quantum mechanical phenomenon in which electrons within the fluorescent molecule are excited to oscillate as a dipole by an externally applied electromagnetic field. Electrons remain in the excited state for a short time, and subsequently relax to their ground state by emission of a photon, which then propagates away as an electromagnetic field [1]. Seen as a dipole antenna for collection and re-emission of electromagnetic energy, a fluorophore is capable of interacting with its immediate environment. A fluorophore's rate of excitation, excitation lifetime, and emission direction can be engineered to achieve much greater detection sensitivity than would be possible for the same emitter attached to a plain glass surface or suspended in solution.

Efficient excitation and recovery of light emitted from fluorescent molecules by employing photonic structures or nano-patterned substrates can result in greatly enhanced signal-to-noise ratio (SNR) fluorescence detection. A high SNR measurement is highly desirable for fluorescence experiments involving fluorophore-tagged analytes at low concentrations. There is now a wealth of literature citing improvements in fluorescence output when metallic surfaces are placed in close contact with a fluorescent system [4–7]. Metal-enhanced fluorescence (MEF) is partly due to a modification of the radiative decay rate of a fluorophore in close proximity to a metallic surface [8,9]. The result is a reduced spontaneous decay time, an increased probability of photon emission, and consequently a higher quantum yield. Metallic nano-islands and colloids support localized surface plasmon resonance and create local uniform local electromagnetic field “hot spots” with which fluorophores can interact and fluorescence with greater intensity, due to enhanced light absorption rate. However, the lossy metallic material dissipates energy from the fluorophores in a direct non-radiative process, known as the metal quenching effect, which leads to reduced fluorescence output [10]. Because maximum quenching occurs within the same locations close to a metal surface where the electric field enhancement is also maximized, MEF methods have not achieved the same scale of enhancement factor that is available from high quality-factor dielectric-based resonators.

Recent research has demonstrated that a nanostructured photonic crystal (PC) optical surface is capable of enhancing the signal of fluorescent dyes by more than two orders of magnitude [11]. In contrast to MEF, the PC enhanced fluorescence (PCEF) provides a consistent and highly efficient platform for enhancement of fluorescent signal by exploiting its optical resonance. A wide variety of PC structures have been studied and fabricated for an enormous range of applications since the periodic nanostructure was first proposed to control the spontaneous emission of materials embedded within the PC by engineering the photonic density of states [12]. Here, the PCs designed for enhanced fluorescence applications are comprised of a periodically modulated low refractive index dielectric surface structure coated with a high refractive index dielectric thin film. Optical resonance in the PC is excited when evanescently diffracted orders couple to modes existing in the high refractive index layer [13,14]. PCs can be engineered to interact strongly with any optical wavelength of interest through selection of their materials and the parameters of their geometry. One-dimensional PCs comprised of stacks of dielectric multilayers have also shown potential for fluorescence enhancement [15].

PCEF takes advantage of PC resonances associated with two phenomena: *Enhanced excitation* and *enhanced extraction*. Enhanced excitation is the result of photons from the illumination source (typically a laser) coupling to a PC resonance. At the wavelength of optical resonance, light will couple to the PC surface from a particular incident angle, resulting in strengthened electric fifrom that can be many times higher than the amplitude of incident radiation. The strong optical near-fields at resonance greatly increase the intensity of the incident laser light near the surface of PCs, yielding amplified output from the fluorophores, due to an enhanced optical absorption rate. The strength of the enhanced excitation effect is directly related to the quality factor (*Q*-*factor* = *Δλ*/*λ*, *Δλ* is the linewidth of the resonance at *λ*) of the PC structure. A high *Q*-*factor* resonance leads to high intensity near-fields where intensity at the PC surface can easily be two orders of magnitude greater than that of the incident radiation [16–19].

The enhanced extraction mechanism involves a spatially and spectrally biased funneling of the emission from surface-bound fluorophores. In a symmetric fashion to enhanced excitation, photons emitted by flurophores near the PC surface can couple to particular optical resonances supported by the PC. The leaky nature of the PC resonant modes warrants the delivery of the emitted photons to propagating waves. Modulated by the PC resonance, the emission of surface-bound fluorophores is no longer omnidirectional, but strongly directed to a narrow set of angles determined by the dispersion of PC [18]. Spatially and spectrally aligning the position of photon detector, the PC resonance, and the emission spectrum of fluorophore, significantly increases the collected signal. The simultaneous implementation of both enhancement features on a PC substrate has been shown to boost the emission from dye molecules by greater than three orders of magnitude [11].

PCEF can be applied to any surface-bound fluorescence assay. Being of particular interest for high-throughput analysis, PCEF was implemented for DNA and protein microarrays. The microarray, consisting of a solid substrate populated with spots of immobilized biological capture molecules, has become an important analytical tool for life sciences research [20]. A microarray may be comprised of thousands or even millions of capture probes that bind to target molecules in a complex biological sample. When a test sample is incubated with the microarray, the analyte molecules can bind specifically to their complementary immobilized ligands. A fluorescent label is added either before or after this binding reaction. The fluorescence from microarray spots is quantified with optical instrumentation to quantify the amount of each specific analyte molecule probed during the assay. Currently, microarrays are performed on glass microscope slides, and are capable of detecting analytes over ∼5 orders of magnitude in analyte concentration, with detection limits typically on the order of 5–10 pg/mL, depending upon the affinity of the capture molecule for its target, and the assay conditions [21,22]. By using PCs as the substrate to perform the microarray assay, we can take advantage of both enhanced excitation and extraction effects to reduce detection limits, to increase SNR for analytes that are near the detection limits, and to enable a detection instrument to operate using lower-cost components. Such a capability will enable disease biomarkers to be detected at lower concentrations for earlier disease diagnosis, and increases the potential for multiplexed biomarker assays to be performed in a clinical setting.

In this review, we begin by describing how specific types of PCs are designed, inexpensively fabricated over large surface areas, and applied to enhance the excitation intensity and emission extraction efficiency. The performance of three types of PCEF detection systems: a confocal laser scanner, a fluorescence microscope using collimated laser illumination, and a laser line-scanning instrument, are compared in Section 3. Among the three detection instrument approaches, the laser line-scanning instrument most effectively optimizes the near-field enhancement capabilities of a one dimensional PC. We also review our efforts towards implementation of PCEF for multiplexed cancer biomarkers and gene expression analysis where PC substrates enable the detection of analytes at lower concentrations than possible without a PC. Finally, we describe new approaches that incorporate the PC resonant surface into external optical cavities that have been demonstrated to further increase fluorescent output. This paper serves as a review for demonstrating design requirements of PC surfaces, methods of fabrication, detection instrument design, and assay capabilities of PCEF. For full details, the reader is directed to full articles published on each topic.

## 2. Design and Fabrication of PC Surfaces for Enhanced Fluorescence

### 2.1. Photonic Crystal Design, Aided by Rigorous Coupled Wave Analysis

**Figure 1.**
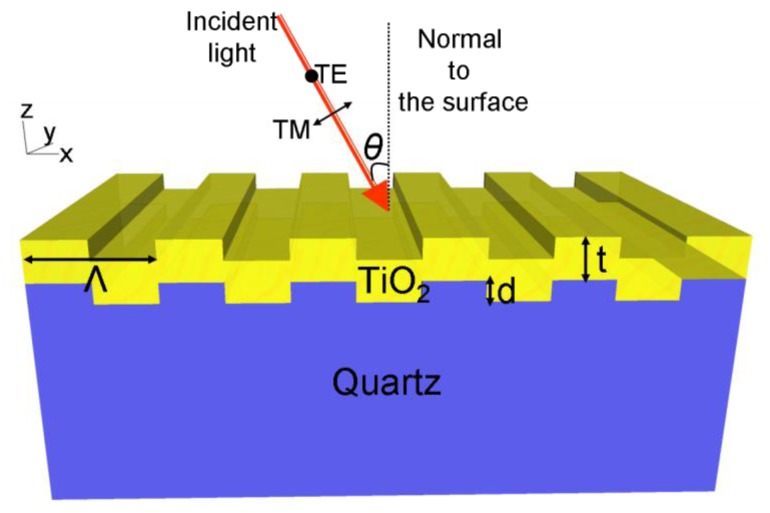
Schematic drawing of a one-dimensional PC surface. The structure is comprised of a low refractive index quartz substrate (*n* = 1.456) containing one-dimensional periodic structure (*Λ* = 400 nm) and coated with a high index layer of TiO_2_ (*n* = 2.35). The physical parameters of the device, refractive indices of the materials and the launch angle (*θ*) of the incident beam combine to determine the resonance wavelength.

A schematic diagram of a one-dimensional PC is shown in Figure 1. The structure comprises a one-dimensional surface structure of height (*d*) and period (*Λ*), that is fabricated from a low refractive index (*RI*) substrate. The structure is coated with a layer of high *RI* material of thickness *t*. The periodic modulation of the grating, which satisfies the second order Bragg condition, allows for phase-matching of an externally incident beam into the resonant modes. The high refractive index material functions as a light confinement layer that supports and intensifies electric field that extends from the device surface into the surrounding medium. This structure supports resonant modes at a specific combination of wavelength and angle of incidence [23]. The near-field intensity associated with the resonant modes is strongly enhanced with respect to the field intensity of the excitation light. The enhanced near fields on the surface of the substrate can amplify the emission intensity from target fluorescent molecules placed on the surface. The wavelength, angle of incidence, bandwidth, and efficiency of the PCEF surface is determined by its geometry as described in previous publications [16,18]. By adjusting the PC geometric parameters, including the grating period (*Λ*), grating depth (*d*), duty cycle (*f*), thickness and refractive index of the dielectric coating, a PCEF surface can be designed to efficiently interact with the absorption and emission spectra of specific fluorescent dye molecules.

A PC structure usually supports two orthogonal modes: transverse electric (TE) polarized and transverse magnetic (TM) polarized. The electric field vector of TM modes are oriented perpendicular to the grating lines of a one dimensional PC structure, while the electric field vector of TE modes is oriented parallel to the grating lines. Compared to TE modes, the resonant modes associated with TM polarization have higher *Q*-factor, resulting in stronger field intensity near the device surface. Therefore, the PCEF surfaces are generally designed to have one TM mode at the excitation wavelength of the fluorophores for near field enhancement, resulting in “enhanced excitation”. The PC can also be designed to have a second resonant mode (TE or TM) that overlaps with the emission spectra of the fluorophores, to direct the emitted photons towards the detection optics to obtain an enhanced extraction effect [18]. Fluorescent output efficiently coupled into the second resonant mode exhibits an angle dependent emission.

**Figure 2.**
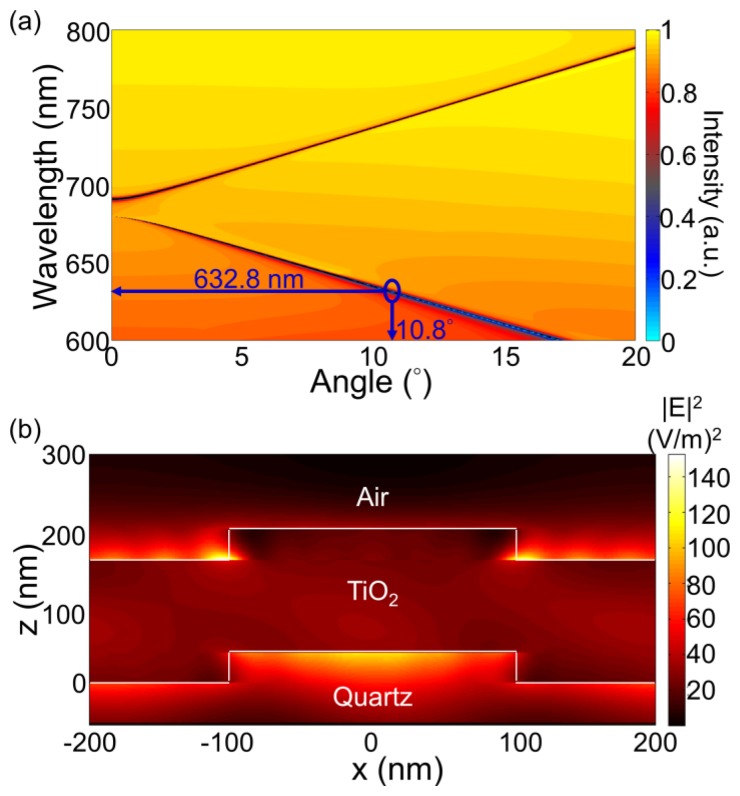
(**a**) Rigorous coupled wave analysis simulated dispersion diagram for the PC shonw in Figure 1. Resonance for the enhanced excitation for the TM mode is at *θ*—10.8° (**b**) Simulated near field distribution at *λ* = 632.8 nm (normalized to the intensity of the incident field) Reprinted from [11] with permission. Copyright 2010 Optical Society of America.

**Figure 3.**
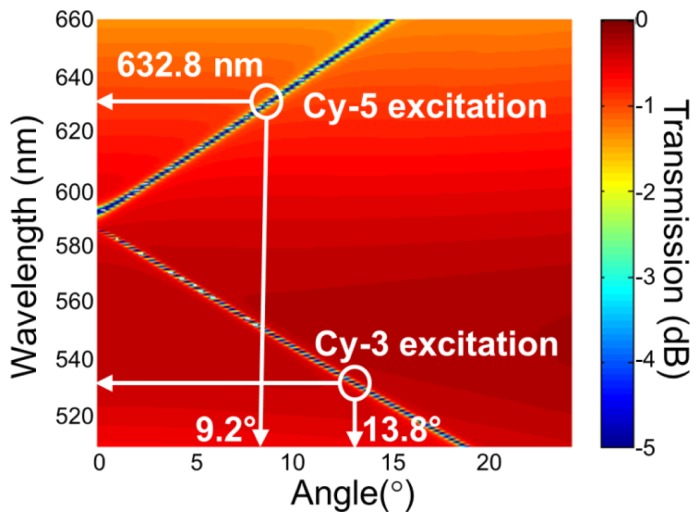
(**a**) Simulated dispersion diagram for the PC used to enhance fluorescence from both Cy-5 and Cy-3 excitation wavelengths. Resonance for the enhanced excitation for the TM mode is *θ*−9.2° for Cy-5 excitation and *θ*−13.8° for Cy-3 excitation. Reprinted from [19] with permission. Copyright 2010 American Institute of Physics.

For the PC structure, transmission efficiency minima (or reflection efficiency maxima) in the simulated far-field diffraction spectra are used to identify the resonant modes. Figure 2(a) plots the photonic band diagram of the PCEF surface. From the photonic band diagram, a resonant angle of *θ_ex_* = 10.8° corresponds to a resonant wavelength of *λ_ex_* = 632.8 nm for enhanced excitation of the fluorophores excited by the He-Ne laser. Figure 2(b) shows the spatial distribution of the simulated near-field electric field intensity (normalized to the intensity of incident field) within one period of the PC structure for resonant wavelength *λ_ex_* = 632.8 nm and resonant angle of *θ_ex_* = 10.8°. The influence of the resonance phenomenon on the resulting near-fields is clearly manifested in the electric field intensity. Similarly, a PC can be engineered to have multiple modes at different resonance angles that can be used to couple different lasers with its resonant modes. Figure 3 plots the band diagram of such a PC that was designed to couple both a green laser (Nd:YAG) at *λ* = 532 nm and a He-Ne laser at *λ* = 632.8 nm. Such a surface can be used to enhance the fluorescence output from various fluorophores that have different excitation wavelengths [19].

### 2.2. Photonic Crystal Fabrication Using Replica Molding on Plastic Substrates and Nanoimprint Lithography on Quartz Substrates

PCs can be inexpensively fabricated over continuous rolls of flexible plastic film using nano-replica molding, thus enabling devices to be economically compatible with single-use, disposable applications [24]. The molding process uses a rigid “master” structure and a photocurable liquid polymer material. Briefly, a silicon master wafer with a negative surface of the desired grating pattern is fabricated using deep-UV lithography and reactive ion etching, typically at a semiconductor foundry in order to obtain precisely defined dimensions and excellent uniformity across a wafer. A liquid UV-curable polymer is then sandwiched between a PET sheet and the silicon master wafer, and is subsequently cured using a high intensity UV lamp. The hardened polymer grating adheres to the PET and is peeled away from the master, enabling a single master to produce thousands of identical replicas. A thin SiO_2_ intermediate layer (*t*_SiO2_—80 nm) is then sputter deposited onto the polymer grating, followed by sputter deposition of the TiO_2_ thin film.

When the PC is illuminated at the resonant coupling condition by a laser, the enhanced electric fields are mainly confined to the surface, but can extend slightly into the media above and below the PC grating, and thus excite fluorescence in material contained in those regions. Because polymer materials, such as those used for replica molding, are slightly fluorescent, the role of the SiO_2_ layer is to insert a nonfluorescent spacer material between the polymer and the enhanced field region of the PC. Because PCEF will enhance the output of any fluorescent emitter within the enhanced field region, one must take care to eliminate all potential sources of autofluorescence, which have the effect of providing a “background” level of light intensity that can potentially overwhelm the intensity of fluorophores that label biological analytes. Potential sources of autofluorescence include the TiO_2_ material, surface chemistry layers, and nonspecific adsorption of fluorescent tags.

Even though nano-replica molding has many advantages for PC manufacturing, detection limits for low concentration biological analytes is limited by the autofluorescence from the plastic substrate and the replica molded polymer grating. It is well known that plastic materials show significant autofluorescence when excited by near-UV or even visible radiation [25–27], with autofluorescence increasing as the illumination source photon energy is increased. This phenomena was demonstrated clearly in a recent publication, in which a PCEF surface designed for multiple excitation wavelengths (*λ* = 532−633 nm) showed best signal-to-noise sensitivity performance for longer excitation wavelengths, but the detection limit for short excitation wavelengths was limited by substrate autofluorescence [19]. Meanwhile, a great deal of research activity is directed towards developing new plastic substrates with lower autofluorescence [28–30]. PMMA, PDMS, Topas and Zeonex have been shown to have lower autofluorescence compared to other plastic materials and have been identified for potential applications in high throughput screening devices that rely on fluorescence detection. Despite these efforts, no polymer material provides autofluorescence comparable to quartz.

**Figure 4.**
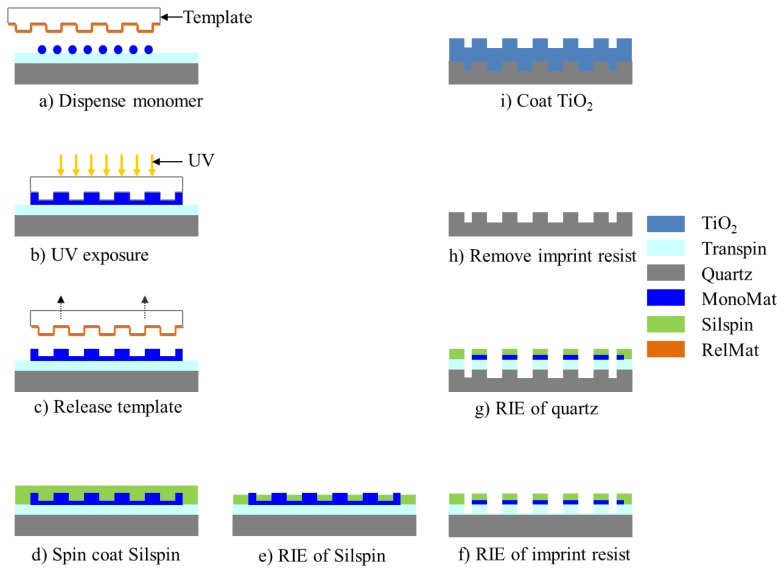
Schematic diagram of the fabrication procedure for producing quartz-based PCs: (**a**) The process begins by dispensing a pattern of MonoMat UV-curable polymer droplets on an unpatterned and polished quartz wafer; (**b**) The template is pressed against the droplets and then UV cured; (**c**) The template is pulled away from the solidified grating pattern; (**d**) A layer of SilSpin imprint resist material is spin coated onto the patterned surface; (**e**) RIE of the imprint resist to expose the quartz surface; (**f**) RIE of quartz to transfer the pattern onto the wafer; (**g**) Piranha cleaning of the wafer to remove the imprint resist residues; (**h**) TiO_2_ deposition onto the grating. Full fabrication details are provided in [11]. Reprinted from [11] with permission. Copyright 2010 Optical Society of America.

**Figure 5.**
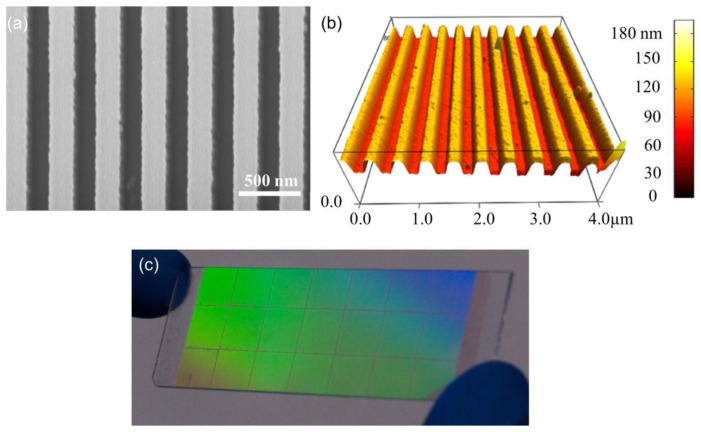
(**a**) SEM image of the top view of the PC fabricated on a quartz substrate by nano-imprint lithography; (**b**) AFM image of the PC, (**c**) Photograph of the PC on 1 × 3 in^2^ substrate. Reprinted from [11] with permission. Copyright 2010 Optical Society of America.

The use of quartz surfaces for PCEF applications has been limited by the requirement to produce surface structures with subwavelength dimensions over surface areas large enough to encompass entire DNA microarrays or protein microarrays, which are usually performed on substrates as large as standard microscope slides (1 × 3 in^2^). Conventional lithography methods, such as e-beam lithography and DUV lithograph are either too expensive or low-throughput to produce subwavelength structures over such large surface areas. In order to address this issue, “step-and-flash” nanoimprint lithography (NIL) tools (Molecular Imprints, Inc., Austin, TX, USA) can be employed to fabricate subwavelength grating structures on quartz substrates [31–34]. SFIL is capable of producing patterns with feature sizes less than 20 nm. In the SFIL process, a template with a pre-defined pattern is pressed into UV-curable liquid over a substrate. Once exposed to UV light, the liquid is cured, and after separation, a replica of the pattern on the template is imprinted into the solidified polymer surface. A step and repeat procedure is used to replicate the pattern over the entire substrate surface. After SFIL patterning, reactive ion etching (RIE) is used to transfer the imprinted pattern into the substrate. Using this approach, we have fabricated one dimensional grating structures with a period of 400 nm upon quartz wafers as large as 8 inches in diameter [11]. Figure 4 outlines the steps of SFIL for fabrication of PCs on low-autofluorescence quartz wafers and Figure 5 shows a large area optical image and SEM of the PC fabricated by SFIL.

## 3. Instrumentation for PCEF

This section will discuss the parameters for efficient excitation of PC resonant modes and the design of detection instruments that have been used for PCEF.

### 3.1. Coupling Condition for 1D PCs

**Figure 6.**
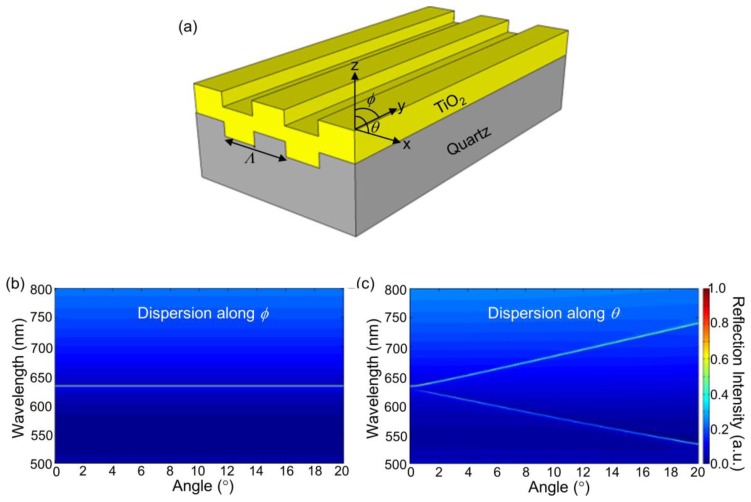
(**a**) Schematic of the PC structure (not to scale). The grating is oriented along the *x*-axis; (**b**) Measured photonic band diagram of the PC surface for *θ* = 0° and *ϕ* varied from 0° to 20°; (**c**) Photonic band diagram of the PC sensor for *ϕ* = 0° and *θ* varied from 0° to 20°.

When a 1D PC is illuminated by a broadband light source, highly efficient reflections from its surface represent the resonance modes at a specific wavelength and a specific angle combination. Figure 6(b and c) show such measured resonant modes for a PC shown in Figure 6(a) when the angle of incidence is scanned from 0° to 20°, both in *n* and *θ* directions respectively (labeled in Figure 6(a)). Here, *θ* is the angle between the incident beam and the grating's normal vector in the plane perpendicular to grating direction and *ϕ* is the angle in the plane along the grating direction. These photonic band diagrams were obtained by illuminating the device with collimated broadband light from a tungsten lamp, and analyzing the reflected light with a spectrometer (USB 2000, Ocean Optics, Denedin, FL, USA) as a function of incident angle. It is evident that the resonant wavelength is not sensitive to the angles along *ϕ* while it changes dramatically when the angle of incidence is varied along the *θ* direction. Using a narrow bandwidth light source, such as a solid-state laser, a misalignment of incidence angle by 0.1° with respect to the resonant angle *θ_R_* will reduce the coupling efficiency of the laser to the PC by a factor of 10. On the other hand, the resonant modes exhibit a very small angular dependence (∼0.3 nm/°) along the *ϕ* direction. Therefore, to efficiently couple light into the 1-D PC surface, the excitation laser beam only needs to be collimated and tuned along the *θ* direction.

### 3.2. Confocal Laser Scanner with Tunable Angle of Incidence

**Figure 7.**
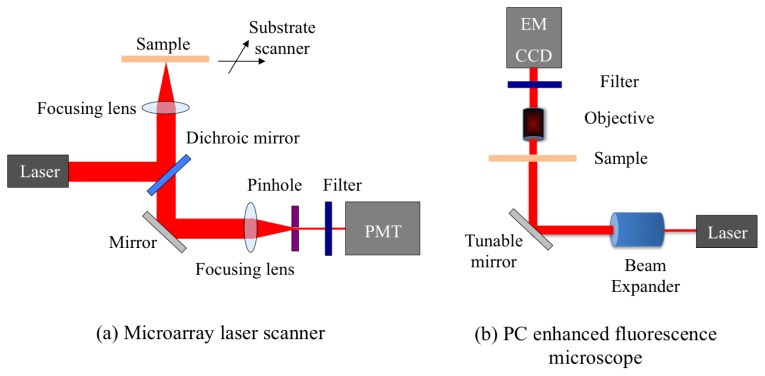
(**a**) Schematic diagram of optical setup of the confocal laser scanner and (**b**) schematic drawing of PC enhanced fluorescence microscope.

A confocal microarray laser scanner (LS Reloaded, Tecan Inc., Mannedorf, Switzerland) was the first detection instrument used to study PCEF. The schematic of the scanner is shown in Figure 7(a). This system is equipped with a He-Ne laser and a solid state Nd-YAG laser as the excitation sources, and a photomultiplier tube (PMT) to detect the emitted fluorescence signal. The angle of incidence of the lasers can be tuned from 0° to 25°. In order to form an image, the substrate is scanned and the fluorescence signal intensity for each pixel is acquired. The instrument uses a lens to slightly focus the laser beam onto the PC, and collects the fluorescence signal resulting from this excitation. Due to the focusing effect, the laser beam has a beam divergence ∼2.5°. Since the beam is not collimated, only a small portion of all the incident light actually matches the resonant mode of the PC, enabling only a fraction of the potential enhanced excitation effect to be utilized.

### 3.3. PC Enhanced Microscope Using Collimated Laser Source

The schematic of a custom-built fluorescent detection system, which is referred to as the PC enhanced fluorescence microscope (PCEFM), is shown in Figure 7(b). In the PCEFM system, the PC fluorescence is imaged by an electron-multiplying charge-coupled device (EMCCD, Hamamatsu Inc., Shizouka Pref., Japan) via a 4 × microscope objective (numerical aperture *N*.*A* = 0.1). Unlike the confocal laser scanner, the PCEFM works in an imaging mode, which significantly improves the throughput for generating an image of a large microarray, as an entire field of view (determined by the magnification of the objective lens) is captured in a single measurement. A He-Ne laser is used as the excitation light source, and a bandpass filter is placed in front of the detector to prevent excitation laser wavelength from reaching the camera.

The PCEFM setup is designed specifically to use a collimated illumination with a tunable angle of incidence. As shown in Figure 7(b), the output of the He-Ne laser is expanded to produce a beam diameter of 20 mm and divergence <0.037° using a beam expander. In order to accurately control the angle of incidence, the PCEFM system utilizes a high-precision angle-tuning gimbal-mounted mirror that is mounted on a motorized linear stage and moves as the mirror rotates. The movement of this linear stage compensates for the beam shift due to any incidence angle variation and thereby ensures a fixed illumination area. The angle tuning resolution of this configuration is 0.005°, enabling one to test PC devices with angular dependence on resonance as narrow as 0.01°. Using this system a coupling efficiency of 98% has been achieved with a PC surface with angular dependence of 0.3° [35].

### 3.4. Objective Coupled Line Scanner for PCEF

It has been shown that the resonant modes of a 1D PC are not sensitive to the changes in angles along *ϕ*. Although the PCEFM does an excellent job at coupling the incident light to the modes of a PC, there is considerable loss in the excitation power in the process of collimating it. In order to solve this problem of loss in power density, a detection approach, in which the excitation source focused by a collimating lens to form a high intensity line, has been demonstrated recently [36]. The goal of this line scanner is to focus light in the *ϕ* direction while maintaining the collimation in the *θ* direction, thus increasing the power density supplied to the fluorescent dye molecules and consequently improve the fluorescent signal strength, while still allowing all the excitation light to couple to the PC resonant mode.

**Figure 8.**
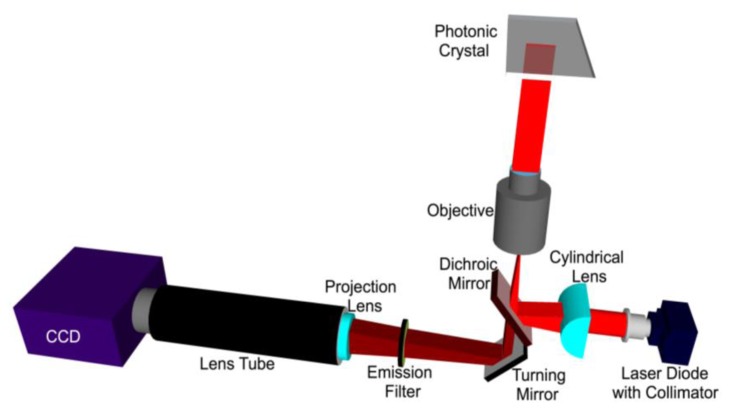
Objective-coupled line-scanning imaging instrument schematic. The incident beam path is represented by a red and the collection beam path is represented in burgundy. The collection and illumination beam paths overlap in the region between the dichroic mirror and the PC.

A schematic diagram of the line scanning configuration is shown in Figure 8. The system is comprised of a 70 mW solid-state laser (AlGaAs) at *λ* = 637 nm coupled to a polarization maintaining fiber, a half wave plate, a cylindrical lens, a long pass dichroic mirror, and a 10 × objective (Olympus Plan N, Center Valley, PA, USA) of focal length 18 mm. The fiber tip is coupled to a fiber collimator giving a highly collimated output beam of 3.4 mm in diameter. The output beam then passes through a half-wave plate, which is used to rotate its polarization to match with the PC-resonant mode to be excited. The laser beam is then focused in the *ϕ* direction to a line by a cylindrical lens (*f* = 100 mm). The focused laser line is then directed onto the back focal plane of the microscope objective via a dichroic mirror. The output of the objective is thus a laser beam focused to a line.

The PC is placed on a motorized sample stage (MS2000, Applied Scientific Instruments, Eugene, OR, USA) that is translated perpendicular to the laser line for a fast scan (750 lines/second). The fluorescence image is constructed by sequential scanning across the sensor in fixed increments. The PC, placed at the focal plane of the infinity corrected 10 × objective (*f_0_* = 18 mm), interacts with a beam that appears collimated in one plane but focused in the other. The theoretically expected line width of a focused beam is given by:

(1) $$w_{0}=\left(\frac{4\lambda }{\pi }\right)\;\left(\frac{f}{D}\right)$$

Here *λ* is the wavelength of the laser beam, *f* is focal length of the focusing lens and *D* is the diameter of the incident beam. The theoretical linewidth of the beam focused on the back focal plane of the objective is calculated to be 23.85 μm. This linewidth is important when characterizing the angle of divergence for the incident beam. The theoretical linewidth in the front focal plane of the objective is calculated to be 4.29 μm. These calculations are subject to the assumptions of a perfectly Gaussian laser beam and perfectly aspherical lenses.

The assembly of the cylindrical lens, half-wave plate and fiber collimator are mounted on a two-dimensional adjustable stage. In order to achieve angle tuning, the line-focused beam is translated on the back focal plane of the objective by tuning the position of the cylindrical lens-wave plate-fiber collimator assembly. This fine stepping is achieved by utilizing a motorized linear stepping stage (Zaber LSM-25, Vancouver, Canada). The result is a change in the incident angle in the *θ* direction. The emitted fluorescence signal is collected by the objective and projected onto a CCD camera (Hamamatsu 9100C) by a tube lens (*f* = 150 mm).

The ultimate goal of the line scanner is to provide illumination that efficiently couples excitation photons to the PC, while providing spatial resolution sufficient for visualization of microarray spots that may be 5–200 μm diameter.

## 4. Utilizing PCEF for Improving the Sensitivity of Microarray Applications

Since their first introduction in the scientific literature in 1982, microarrays have revolutionized the study of gene expression. Microarray analysis has extended to the high throughput study of a host of other biological analytes such as proteins, peptides, tissue, cells, antibodies, and chemical compounds. In our work, we have demonstrated significant gains in detection sensitivity by applying the PCEF technology to the microarray platform in the context of DNA and protein microarrays.

### 4.1. PCEF for Differential Gene Expression Analysis

The DNA microarray gained prominence as a fluorescence-based tool for the high throughput quantification of gene expression, allowing a large number of candidate genes to be examined for differential expression simultaneously without extensive prior knowledge of gene functions. Eukaryotic gene expression is typically characterized by a large number of genes expressed at very low levels and a decreasing number of genes expressed at high levels [37,38]. Usually the fluorescence intensity of only high expression genes—A small fraction of all genes in a cell population—Can be detected above the noise in the experiment. However, the profiling of low expression genes, many of which have important housekeeping functions, is also of keen biological interest. To better quantify these low abundance genes, we began by pursuing the engineering and fabrication of surface PCs on plastic substrates. The nanoreplica fabrication process produces PCs with uniformity over larges areas necessary for high density microarrays comprised of thousands of capture probes. The PCs were fabricated in the format of standard microscope slides, allowing them to be seamlessly incorporated into the standard assay workflow alongside control glass slides, and imaged using a commercially available fluorescence microarray scanner.

To facilitate the binding of DNA molecules to the PC, the slides are functionalized with a vapor-based epoxysilane-based surface chemistry [39] and each PC was paired with a silanized glass microscope slide that acted as the control sample. A set of 192 oligonucleotides representative of soybean genes [40] were printed in replicates of 40, for a total of 7,680 spots per slide; the spots were incubated and then UV crosslinked. Total RNA was extracted from freeze dried soybeans seeds (*Glycine max* cultivar Williams), purified and labeled with Cy5 by reverse transcription. Printed slides were blocked with bovine serum albumin and then hybridized overnight at 42 °C with the labeled samples. Approximately 40 μg of total RNA was used per slide. All slides received identical treatments throughout the assay. All slides were scanned using a confocal microarray scanner (LS Reloaded, Tecan) using a TM polarized laser (*λ* = 632.8 nm) and an emission filter with a range of 670–710 nm. All slides were scanned at identical PMT gain settings and at a pixel resolution of 10 μm. Glass slides were scanned at normal incidence (0 degrees) while the PCs were scanned at their respective resonant angles (*θ_excitation_*). Spot signal-to-noise ratio (SNR) was calculated as the local background subtracted spot intensity divided by the standard deviation of the local background.

The overall effect of the PC enhanced fluorescence phenomena is to amplify the fluorescent signal from molecules within approximately 100 nm of the PC surface. This PC was engineered to enhance the common microarray dye cyanine-5 (Cy5) by more than one order of magnitude when scanned in a commercial microarray scanner [41]. The application of this PC design to a 1-color microarray experiment was pursued where the differential expression was assessed between *Glycine max* cotyledons and trifoliates, which represent tissues from two distinct developmental stages in the soybean plant [42].

**Figure 9.**
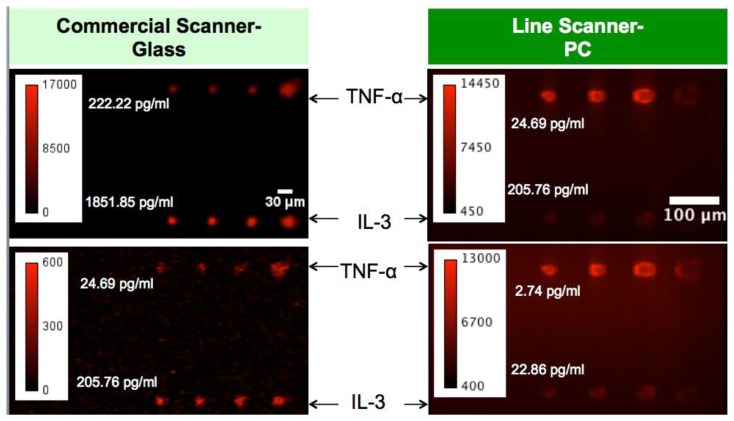
Fluorescent images of microspots from a sandwich immunoassay (TNF-α and IL-3) for two different concentrations obtained using the objective coupled line scanning system.

The signal enhancement factor is defined as spot intensity subtracted by the local background observed on the enhancement substrate divided by the same value observed on the glass slide or control substrate. The signal enhancement factor observed from Cy-5 spots with high expression genes in this microarray experiment was approximately 60 × (see Figure 9).

**Figure 10.**
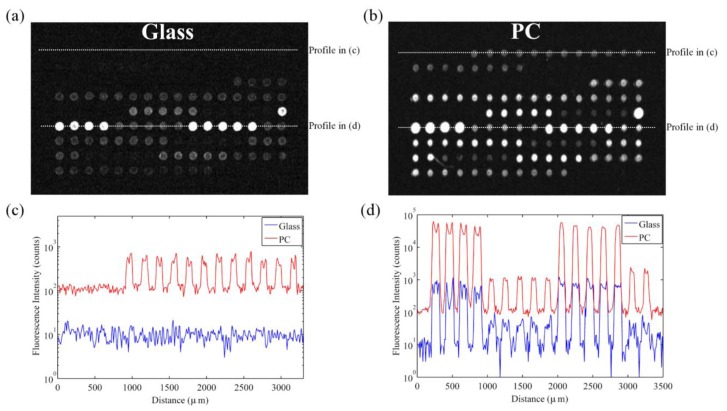
Representative fluorescence images of an identical subarray on (**a**) the PC and (**b**) glass slide. Both images were taken at identical gain settings. Line profiles through low expression genes (**c**) and high expression genes (**d**) provide a comparison of spot intensities on the PC and glass slides. Reprinted from [42] with permission. Copyright 2010 ACS Publications.

However, SNR enhancement is a more relevant measure over signal enhancement because microarray data analysis programs use SNR values to classify spots as detected or not detected. It is possible to achieve good signal enhancement without achieving similar SNR enhancement if a substrate enhances fluorescence but also has a large noise value, thus avoiding any advantages of fluorescence enhancement. We observe that the PC not only attains a large signal enhancement but it also achieves an SNR enhancement of approximately 10 × (measured over all spots in the experiment), suggesting that the array can detect hybridization at concentrations 10 × lower than can be detected on glass substrates. Since the SNR is enhanced by more than one order of magnitude on PC substrates, genes with expression levels that were lower than the noise floor on glass substrates can now be measured on PCs. This allows researchers to preserve the advantageous throughput of microarrays while increasing the sensitivity of their measurements. The practical effect of the PC is to improve the dynamic range of the expression measurements and allow for quantification of low expression genes. The direction of differential expression in these low expression genes is confirmed by sequencing data, which agreed with the microarray analysis for 39 of the 41 genes identified as differentially expressed only on PC microarrays. By expanding the dynamic range of the microarray experiment, the number of genes for which statistically significant changes in expression could be observed improved from 26 to 66 genes, or from 13% to 34% of the genes probed in the experiment. This suggests that the detection capabilities of current microarray protocols can be greatly expanded by just the substitution of conventional substrates with an enhanced fluorescence substrate like the PC. By enhancing fluorescence, more than double the number of genes were identified on the PC as differentially expressed between the trifoliate and cotyledon tissues, demonstrating that enhanced fluorescence offers practical benefits to a DNA microarray experiment (Figure 10).

Utilizing a PC surface amplifies the fluorophore intensity relative to the autofluorescence from the plastic substrate; however the impact of substrate fluorescence on the measurements, mainly from the replica molding material, will mask the detection of very low fluorescence intensity from the analyte. Substrate auto-fluorescence is an important contributor to the experimental noise in a microarray, making the choice of this material very critical. To address this concern, surface PCs fabricated on low-autofluorescent quartz substrates were adopted. The PC used here is composed of a one-dimensional periodic structure formed on a low refractive index quartz surface that is coated with a high refractive index layer of TiO_2_.

In the same DNA microarray experiment on quartz based PCs and control glass slides where the expression profile of a *Glycine max* cotyledon sample was studied, the impact of spot intensity enhancements resulted in a similar increase in the number of genes that were detectable on the PC as compared to glass [43]. On average, twice as many genes were detectable on the PC. Many of the highly expressed genes, detected both on the glass and PC, encode storage proteins which are abundant during this stage of seed development. The additional genes detected only on the PC represent enzymes and important regulatory transcription factors that are expressed at lower levels. Transcription factors provide genetic control over development and can also be important markers of disease state. The ability of the PC to detect these low abundance transcripts is reflected by the lower average fluorescence intensity of 515 counts for genes detected only on the PC as compared to an average fluorescence intensity of 3,280 counts for genes detected on glass.

Although the gains from the fluorescence enhanced afforded by the quartz PCs in the context of these DNA microarrays are comparable to that of the plastic based PCs, the quartz PCs offer a unique advantage. Substrate autofluorescence is an important source of noise in a microarray experiment, and increases when illuminated with higher energy (or lower wavelength) photons. The quartz PCs have a substrate fluorescence that is 15 times lower than plastic PCs. This is an important consideration when designing a PC to enhance fluorescence emission from cyanine-3 (Cy-3, *λ_excitation_* = 536 nm), another fluorophore that is routinely used in DNA microarrays. Devices fabricated on low-fluorescence quartz substrates and designed for multiple fluorophore enhancements have been pursued for 2-color DNA microarray applications.

The increased SNRs provided by the PCs may allow researchers to perform experiments that are currently problematic on glass slides. Because lower amounts of bound sample can be detected with the PC, sample sizes may be reduced to volumes that would be difficult to probe using normal glass substrates. This may be particularly helpful for profiling gene expression in limited tissue samples or small populations of rare cells. This approach is not limited to conventional DNA microarray experiments. Any surface-bound biomolecular assay can be performed on these PCs for improved performance, as we will next illustrate with protein microarrays.

### 4.2. PCEF for Multiplexed Protein Biomarker Detection

Antibody-based protein microarrays are a valuable tool for studying cellular protein production with potential applications as a clinical tool in disease diagnosis and drug discovery [44–47]. Protein microarrays are a favorable platform for the detection of circulating biomarkers because they combine multiplexed detection, minimal reagent usage, and high sensitivity. By running calibration standards alongside patient samples, protein microarrays provide quantitative measurements of analyte concentration. Furthermore, protocols have been developed that demonstrate multiplexed detection of biomarkers in serum through fluorophore-tagged secondary antibodies. In many clinically relevant applications, such as for detection of biomarker proteins that are expressed by cancer cells at a tumor site and subsequently diluted by the total blood volume of a person, a target protein may only be present at concentrations in the 1–100 pg/mL range [48–51]. There is substantial interest in extending the limits of detection (LOD) and generally increasing the SNR in order to diagnose disease at the earliest possible stage and to quantify biomarker levels at concentrations that were below previous limits of detection. Fluorescent-based detection of chemically tagged analytes has been demonstrated as a robust, highly specific, and easily multiplexed method for achieving high sensitivity [52–57].

We have utilized PCEF to develop a high-sensitivity platform for the detection of a panel of >20 breast cancer biomarkers in a protein microarray format [58,59]. Our results show that the resonant excitation effect increases the signal-to-noise ratio by 3.8- to 6.6-fold, resulting in a decrease in detection limits of 6–89%, with the exact enhancement dependent upon the antibody-antigen interaction. Dose-response characterization of the photonic crystal antibody microarrays demonstrates the capability to detect common cancer biomarkers in the <2 pg/mL concentration range within a mixed sample using polymer-based PCs and a commercially available confocal microarray laser scanner.

**Figure 11.**
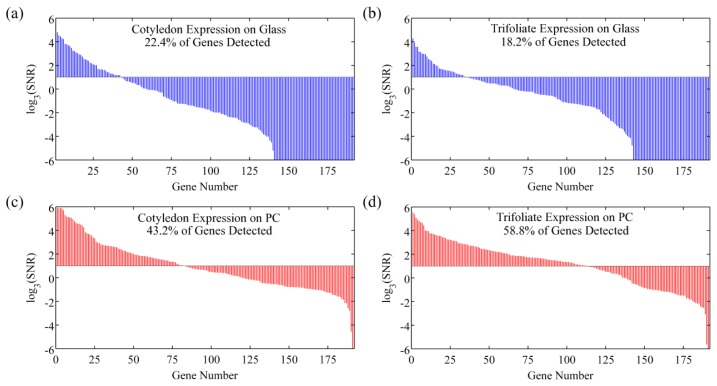
Replicate-averaged SNR values for 192 genes probes are presented in a logarithmic plot. Genes are ordered by their average SNR value and a cutoff line of SNR = 3 is used in each graph to determine the number of genes detected. Cotyledon expression profiles are plotted in (**a**) for a glass slide and (**c**) for a PC slide, respectively. Trifoliate expression profiles are presented in (**b**) for a glass slide and (**d**) for a PC respectively. Reprinted from [42] with permission. Copyright 2010 ACS Publications.

The first step in the assay is to print capture antibodies on the slide; replicate arrays are printed to assess the experimental variability in each slide. The slide is blocked to limit the non-specific binding of analytes in subsequent steps. The slide is then incubated with a test sample consisting of a mixture of biomarkers. Next, the slide is washed to remove all unbound biomarkers and then incubated with a mixture of biotinylated secondary detection antibodies. Finally, the secondary detection antibodies are labeled by incubation with streptavidin-Cy5 (Figure 11). In this platform, alongside the test samples, it is routine to generate a standard curve by assaying a concentration series that covers a 10,000-fold range of protein concentrations.

The effect of PC enhanced fluorescence was determined by comparing the fluorescence output when the PC is illuminated with the excitation laser at an incident angle matched to the PC resonant angle (“on resonance”, in this case 0°) to the fluorescence output when the illumination angle is not at the PC resonance (“off resonance”, in this case 20°).

**Figure 12.**
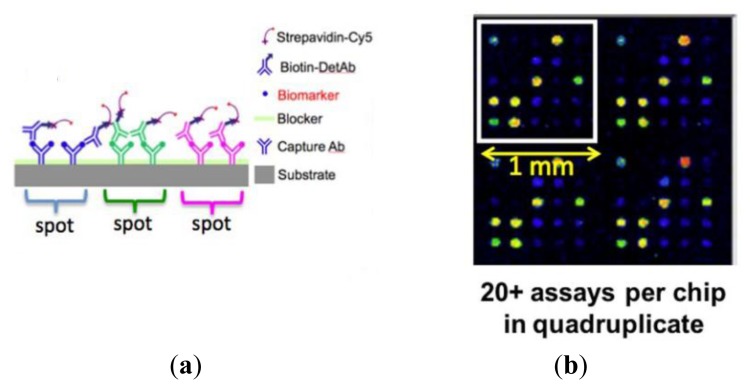
(**a**) Schematic diagram of the protein microarray assay format. Each microscope slide is divided into 16 wells and each well consists of 20 capture antibodies printed in quadruplicates; (**b**) A fluorescence image of a well obtained at the completion of the assay; spot color is representative of biomarker concentration.

The fluorescent image of one block of an array exposed to the second highest concentration in the dilution series depicts the typical signal enhancement observed when the PC is imaged on-resonance. The fluorescence signal intensity was enhanced by a factor of 11 to 20-fold by illuminating the PC at its resonant condition (Figure 12), using the commercially available confocal microarray scanner described in Figure 7(a).

**Figure 13.**
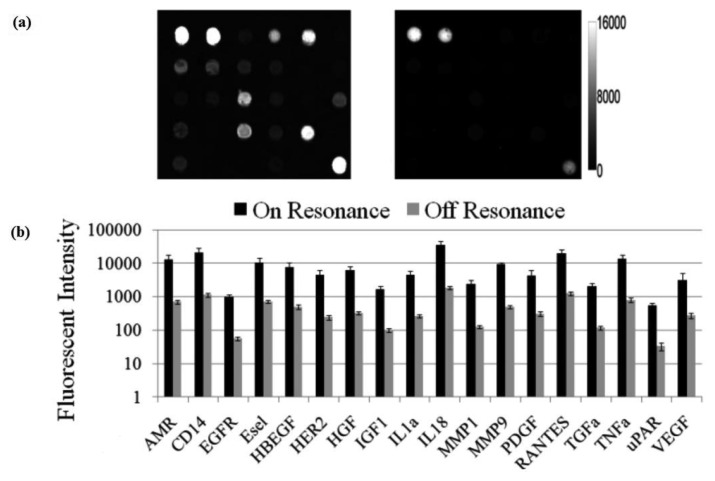
(**a**) Fluorescence images of Cy5 labeled protein microarray spots from a block exposed to a mix of 20 biomarkers. Images on the left and right indicate the block on the PC scanned an resonance and off resonance, respectively. Images were obtained with a confocal microarray laser scanner and at identical gain settings; (**b**) Comparison of the replicate-averaged fluorescence intensity at PC resonance and off PC resonance for all functional assays in the array. Error bars indicate +/− one standard deviation of 8-replicate spots. Reprinted from [58] with permission. Copyright 2011 ACS Publications.

**Figure 14.**
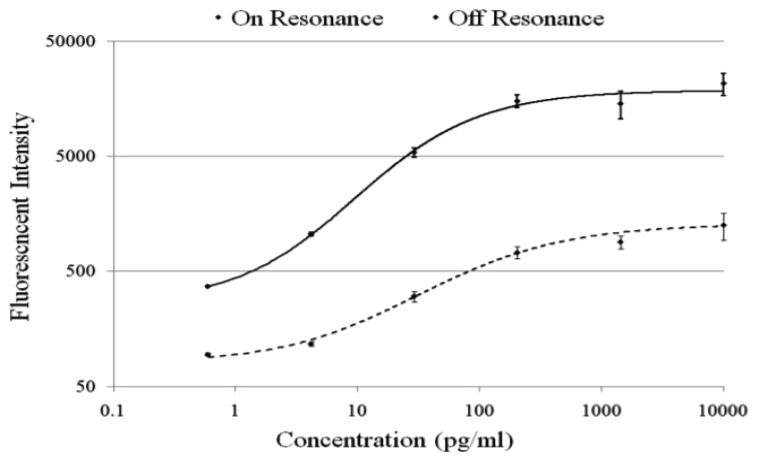
Standard curves for TNFα when the PC is imaged at its resonance condition (solid curve) and off resonance (dashed curve). The PC demonstrates higher detection sensitivity at its resonance condition as indicated by the steeper slope in the linear region of the solid curve. Sensitivity here is defined as the fluorescence signal changer per unit change in concentration. Reprinted from [58] with permission. Copyright 2011 ACS Publications.

The PC enhances the output of any fluorophore within the evanescent field region, regardless of whether the source of the fluorescence is a Cy5 molecule within the capture spot area, a Cy5 molecule nonspecifically bound outside the capture spot area, autofluorescent material within the device structure, or autofluorescence from the chemical functionalization layer. It is observed that when the PC is on-resonance, the background intensity is 4 to 5-fold higher compared to the off-resonance condition. Even so, an overall SNR enhancement of 3.8- to 6.6-fold was observed for the assays because the magnitude of the PC enhancement is greater within the capture spots than in the regions between the spots. Observed enhancements in SNR is particularly important for detecting antigens at low concentrations—For example, two antigens EGFR and uPAR were detected at concentrations as low as 3.6 and 7.1 ng/mL, respectively. At the PC off resonance, the spot signals for EGFR and uPAR at these same concentrations were noise-limited (SNR < 3) and could not be differentiated from the local background fluorescence. However, at the PC resonance, these same spots were detectable (SNR > 8) over the background. The ability to detect reduced concentrations of such antigens is extremely important to the early detection of disease biomarkers, which in general are present at very low concentrations in serum. The signal intensities from each dilution in the concentration series were used to generate standard curves using the Protein Microarray Analysis Tool (ProMAT) software, developed by Pacific Northwest National Laboratory. A representative standard curves for TNFα when the PC is on-resonance and off-resonance is presented in Figure 13. We found that when on-resonance, the PC demonstrated better precision as indicated by the steeper slope in the linear region of the standard curves, and ∼10-fold reduced limit of detection. Dose-response curves for detection of one biomarker (TNFα) is shown in Figure 14, with measurements taken on-resonance and off-resonance, in which the enhanced excitation effect provides a clearly amplified signal for all concentrations.

## 5. Conclusions

As shown in the preceding examples, PC surfaces offer important advantages in fluorescence-based molecular diagnostic applications such as gene expression analysis and biomarker-based disease diagnostics by providing the ability to amplify the number of emitted photons compared to performing the same assay upon unpatterned glass surfaces. Composed of dielectric materials, PCs can easily replace glass substrates in current fluorescence-based assays without modifying surface chemistries involved. They can be inexpensively and uniformly fabricated over large surface areas using plastic substrates or ultra-low fluorescence quartz substrates. PCs are compatible with commercially available confocal laser scanners; but for optimal performance, a specially adapted fluorescence microscope with collimated laser excitation source is most desirable. In order to further improve the fluorescence on PC surfaces, next generation PC enhanced fluorescence detection techniques are being developed [60,61]. As the detection capabilities of PCs improve, it will enable researchers and clinicians to assay lower concentration biomarkers while preserving the high-throughput microarray format. Besides surface bound fluorescence assays, PCEF can be extended to a microfluidic channel, enabling enhanced detection of analytes (such as virus particles or DNA molecular beacons) flowing through the channel and entering the evanescent field region of the PC [62].
